# A Subpopulation of Circulating Endothelial Cells Express CD109 and is Enriched in the Blood of Cancer Patients

**DOI:** 10.1371/journal.pone.0114713

**Published:** 2014-12-15

**Authors:** Patrizia Mancuso, Angelica Calleri, Giuliana Gregato, Valentina Labanca, Jessica Quarna, Pierluigi Antoniotti, Lucia Cuppini, Gaetano Finocchiaro, Marica Eoli, Vittorio Rosti, Francesco Bertolini

**Affiliations:** 1 Laboratory of Hematology-Oncology, European Institute of Oncology, Milan, Italy; 2 Department of Neuro-Oncology, Fondazione IRCCS Istituto Neurologico C. Besta, Milan, Italy; 3 Center for the Study and Treatment of Myelofibrosis, Research Laboratories of Biotechnology, Fondazione IRCCS Policlinico San Matteo, Pavia, Italy; University of Liverpool, United Kingdom

## Abstract

**Background:**

The endothelium is not a homogeneous organ. Endothelial cell heterogeneity has been described at the level of cell morphology, function, gene expression, and antigen composition. As a consequence of the genetic, transcriptome and surrounding environment diversity, endothelial cells from different vascular beds have differentiated functions and phenotype. Detection of circulating endothelial cells (CECs) by flow cytometry is an approach widely used in cancer patients, and their number, viability and kinetic is a promising tool to stratify patient receiving anti-angiogenic treatment.

**Methodology/Principal Findings:**

Currently CECs are identified as positive for a nuclear binding antigen (DNA+), negative for the pan leukocyte marker CD45, and positive for CD31 and CD146. Following an approach recently validated in our laboratory, we investigated the expression of CD109 on CECs from the peripheral blood of healthy subject and cancer patients. The endothelial nature of these cells was validated by RT-PCR for the presence of m-RNA level of CDH5 (Ve-Cadherin) and CLDN5 (Claudin5), two endothelial specific transcripts. Before treatment, significantly higher levels of CD109+ CECs and viable CD109+CECs were found in breast cancer patients and glioblastoma patients compared to healthy controls, and their number significantly decreased after treatment. Higher levels of endothelial specific transcripts expressed in developing endothelial cells CLEC14a, TMEM204, ARHGEF15, GPR116, were observed in sorted CD109+CECs when compared to sorted CD146+CECs, suggesting that these genes can play an important role not only during embryogenesis but also in adult angiogenesis. Interestingly, mRNA levels of TEM8 (identified as Antrax Toxin Receptor1, Antrax1) were expressed in CD109+CECs+ but not in CD146+CECs.

**Conclusion:**

Taken together our results suggest that CD109 represent a rare population of circulating tumor endothelial cells, that play a potentially useful prognostic role in patients with glioblastoma. The role of CD109 expression in cancer vessel-specific endothelial cells deserves to be further investigated by gene expression studies.

## Introduction

Angiogenesis plays a crucial role in tumor growth and progression [1]–[3] and based on the theory that targeting of endothelial cells may be a more effective strategy than targeting tumor cells, during the last decade many anti-angiogenic drugs have been introduced to the clinical setting [4]–[8].

In order to assess circulating biomarkers of angiogenesis that may predict outcome to antiangiogenic therapies in cancer patients, many approaches have been tested in both preclinical and clinical studies [9]–[11] and among these the quantification of circulating endothelial cells (CECs) by flow cytometry has found wide application [12]–[14].

CECs are mature endothelial cells released from vessels during physiological endothelial turnover or, in cancer patients, from the tumour vasculature, where they likely reflect endothelial damage or dysfunction. These cells are increased in cancer patients when compared to healthy subjects, and their modifications in number and viability has shown predictive, prognostic, dynamic or escape biomarker value [15]–[18].

The endothelium is not a homogeneous organ. Endothelial cell heterogeneity has been described at the level of cell morphology, function, gene expression, and antigen composition [19]. As a consequence of the genetic, transcriptome and surrounding environment diversity, endothelial cells from different vascular beds have differentiated functions and phenotype [20]–[24], [35].

Currently endothelial markers used to identify CECs are CD34, CD31 and CD146 in combination with CD45, to exclude leucocytes, and a nuclear staining marker (like Syto16, Hoechst or DRAQ5) to eliminate counting of noncellular endothelial microparticles [14], [25]–[26].

The CD109 gene encodes a glycosyl-phosphatidylinositolanchored glycoprotein that is a member of the alpha (2)-macroglobulin/C3, C4, C5 family of thioester-containing proteins [27]. CD109 interacts directly with the type I transforming growth factor -beta (TGF-β) signaling receptor and negatively modulates TGF-β signalling [28]. It is expressed on a subpopulation of CD34+ cells [29], on activated platelets and activated T-cells [30] and on a subpopulation of endothelial cells [31]. Interestingly, it has been reported that CD109 is one of 12 endothelial markers over-expressed in tumor endothelial cells [23], [24]. In order to gain a more comprehensive understanding of CEC phenotype, we investigated the expression of CD109 on cultured endothelial cells and on CECs from the peripheral blood of healthy subject and cancer patients. We used a flow cytometry approach validated in our laboratory, and the endothelial nature of these cells was validated by RT-PCR and gene expression.

## Materials and Methods

### Ethics Statement

All patients gave written informed consent before inclusion in the therapeutic protocol and for research purpose. All clinical investigation were conducted according to the principles expressed in the Declaration of Helsinki.

For Breast Cancer patients: The trial was conducted at the European Institute of Oncology, Milan, Italy and approved by the “IRCCS - Istituto Europeo di Oncologia e Centro Cardiologico Monzino” Ethic Committee and registered in the Institute database (# 2007-006025-27).

For Glioblastoma patients: The trial was conducted at the Neurological Institute “Carlo Besta” of Milan, Italy and approved by the “ Regione Lombardia Sezione Fondazione IRCCS Istituto Neurologico Besta” Ethic Committee and registered in the Institute database (#1/08).

### Detection of CD109 on endothelial colonies

To validate by flow-cytometry the expression of CD109 on endothelial cells, in vitro endothelial colonies were obtained from peripheral blood (PB) of 10 healthy donors (7 female, 3 male) and 23 cancer patients as previously described [32].

Briefly, mononuclear cells (MNCs) were isolated from PB using Ficoll-Paque gradient centrifugation, resuspended into EGM2 medium (Lonza, Walkersville, MD) and seeded onto Collagen I petri dishes (35 mm, Biocoat, BD Labware, Bedford, MA). Cultures were incubated at 37°C, 5% CO2, 95% relative humidity for 3–4 weeks. Medium was changed every 2 days for 7 days and then twice a week until first passage, and culture monitored for the detection of endothelial colonies on the basis of morphological features, as previously described [32]. After 15–20 days, cells were detached with trypsin/EDTA (Gibco, BRL, UK) and stained with monoclonal antibodies (MoAbs) anti- CD31-PeCy7, CD34-APC, CD45-H7, and 7-AAD (all from Beckman Coulter) CD146-Pe (Chemicon), VEGFR-2- PE (R&D) and CD109-Pe (Abcam) for flow cytometry studies.

### Sorting of CD109+ and CD146+ cells from Peripheral Blood

To perform a microarray analysis for genes expressed in circulating endothelial cells positive for CD109 (CD109+CECs) and CEC positive for CD146 (CD146+CECs), a three laser Influx high speed cell sorter (BD) was used.

Briefly, in two different experiments, 150 ml of blood from 8 healthy subjects were collected and after MNCs isolation with Ficoll- gradient, cells were depleted of white blood cells using anti-CD45 antibody coupled with magnetic MACS MicroBeads (Miltenyi Biotech) following the manufacturer's instructions. Cells were stained for Syto16, CD45, CD31 and CD109 and in the second experiment for Syto16, CD45, CD31 and CD146.

During sorting procedure, samples were continuously cooled to 4°C and a forward scatter pulse height and side scatter analyses were performed to exclude cell clusters and doublets. A two way cell sorting procedure was performed with a140 um nozzle with a 5.5 PSI pressure, and with an events rate of 1,000–1,500 events per second, using a sort pure mode. The sorting gate was painted on Syto16+CD45−CD31+CD109+ cells and on Syto16+CD45−CD31+CD146+ cells. Samples were collected into sterile polypropylene tubes containing RPMI and used for molecular analysis. Purity was always greater than 95% with a recovery of 70–80%.

### RT-PCR and gene expression analysis of sorted CD109+ CECs cells and CD146+ CECs

RNA isolation of sorted CD109+CECs and CD146+CECs, was carried out using ArcturusPicoPureRNA Isolation Kit, and cDNA was generated from the total amount of RNA using the high-capacity cDNA reverse transcription kit (Applied Biosystems). Quantitative real-time PCR (qRT-PCR) was carried out after pre-amplification of cDNA (TaqManPreAmp Master Mix Kit) with an ABI Prism 7000 platform using TaqMan Gene Expression Assay for CDH5 (Ve-Cadherin), CLDN5, (Claudin 5) VWF (Von Willebrand Factor), CD34, VCAM-1, CLEC14a, TMEM204, ARHGEF15, GPR116, ARAP3, TEM8 (ANTRAX1 receptor) [33].

### Characterization of CD109+ CEC and CD146+ CEC phenotype

To characterize CD109+ CEC and CD146+ CEC phenotype, a combination of 10 monoclonal antibodies was used (Syto 16 FITC, CD146 PE, 7-AAD, CD31 PeCy7, CD34 ECD, CD13 APC, CD90 APC-AF700, CD117 APC-AF750, CD45 Pacific Blue and CD109 Pacific Orange). CD90 and CD 117 were purchased by Beckman Coulter.

Unlabeled CD109 (Abcam) was conjugated with pacific orange, using the Zenon ^R^ Kit labeling technologies (Life Technologies), according to the manufacturer instructions.

CD34, CD90 and CD117 expression were evaluated on DNA+ (Syto16+) CD45−CD31+ CD109+ and on DNA+ (Syto16+) CD45−CD31+CD146+ cells.

To confirm by flow cytometry the endothelial nature of CD109+CECs and CD146+CECs, we used Ulex Europeaus lectin and Ac-LDL FITC up take.

For Ac-LDL FITC up take, we collected 5 mL of blood from 5 different breast cancer patients, and after MNCs isolation with Ficoll-gradient, cells were incubated with Ac-LDL FITC according to the manufacture's instruction (10 µg/mL for 4 hours at 37°C). After Ac-LDL incubation, we stained cells with Syto16, CD45, CD31 and CD109 to investigate the presence of AcLDL+CD109+ cells among CD45-Syto16+(nucleated) CD31+ cells. Five different experiments were performed.

### Detection of CD109+ CECs and CD146+ CECs in Healthy Subjects and Cancer Patients

**Figure 1:**
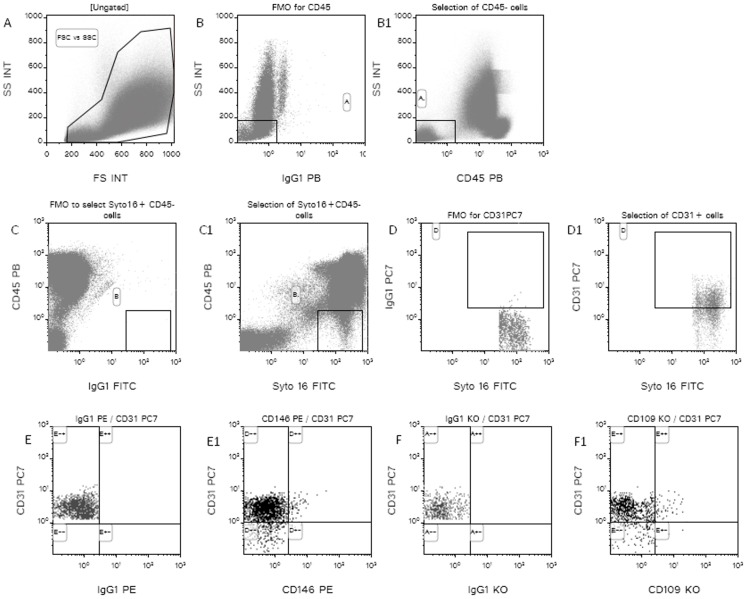
Flow cytometry strategy to detect CD109+CECs and CD146+CECs. Fluorescence Minus One for each fluorescence is reported.

Following a flow-cytometry protocol validated in our laboratory for CEC detection (Fig. 1), [ref 14], the number and viability of CD109+ CECs and CD146+ CECs were evaluated in the peripheral blood of 50 healthy subjects and 200 cancer patients (66 metastatic breast cancer and 134 glioblastoma patients).

Briefly, the nuclear staining Syto16 was used to discriminate between DNA containing cells, platelets and cell debris, and 7AAD to determine the viability status of the cells. The panel of MoAbs used included anti-CD45 (to exclude hematopoietic cells), anti-CD31 (an endothelial cell differentiation marker), anti-CD34 (as endothelial and progenitor cells marker) and anti-CD109 or anti-CD146.

After incubation of 2.5×10^6^ total cells with MoAbs (4°C for 20 minutes) and cells lysis with ammonium chloride (NH_4_Cl), at least 2×10^6^ total cells were acquired at flow-cytometer (Facs-Canto, BD, from 2007 to 2010 and Navios, Beckman Coulter, from 2010 until February 2013). Analysis was performed on cells positive for Syto16 (DNA) negative for CD45, and positive for CD31 and CD109 or for CD31 and CD146.

The combination of Syto16 and 7AAD was used to evaluate CEC viability according to van der Pol et al. [34]. Necrotic cells were identified as Syto16 low/7AAD positive, apoptotic cells as Syto16 low/7AAD negative and viable cells as Syto16 bright/7AAD negative.

### Statistical Analysis

Continuous variables are reported as mean ± SEM. Two-way ANOVA, or two-tailed Student's t test, were used where appropriate. Statistical significance was taken at p<0.05.

## Results

### Endothelial colonies phenotype

Peripheral blood MNC (30×10^6^ cells plated) generated endothelial colonies from 9 of 10 healthy subjects and from 14 of 23 patients. All endothelial colonies were positive for CD31, CD146, CD34 and VEGFR-2 and negative for CD45.

Endothelial colonies from all healthy subjects and from 10 cancer patients were also evaluated for CD109 expression. CD109 was expressed on 1/9 endothelial cultures from healthy subject (11%) and on 5/10 (50%) endothelial cultures from cancer patients.

### RT-PCR of sorted CD109+ CECs and CD146+ CECs

**Figure 2:**
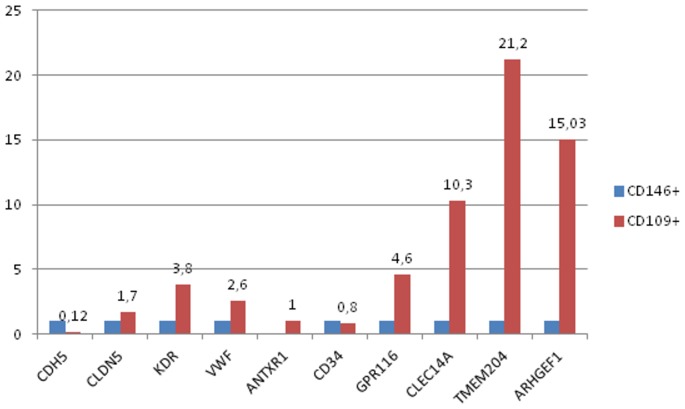
Real-time quantitative PCR analysis comparing changes in gene expression between CD109+ cells and CD146+ cells. Fold changes of down- or up-regulated genes are expressed comparing cDNA from CD109+ to CD146+ cells as reference cells. To ensure biological reproducibility, blood for cDNA collection were obtained from 8 different donors.

Fold changes of down- or up-regulated genes are expressed comparing cDNA from CD109+CECs to CD146+ CECs as reference cells (Fig. 2).

In both CD109+CECs and CD146+CECs we confirmed the expression of mRNA level of CDH5 (Ve-Cadherin) and CLDN5 (Claudin V), two endothelial specific transcripts, suggesting that both cells are related and of endothelial nature [23]–[24]. Same results were obtained for mRNA levels of vWF, VCAM1 and CD34.

Higher levels of endothelial specific transcripts expressed in developing endothelial cells [33] CLEC14a, TMEM204, ARHGEF15, GPR116, were observed in sorted CD109+CECs when compared to sorted CD146+CECs.

Interestingly, mRNA levels of TEM8 (identified as Antrax Toxin Receptor1, Antrax1) were expressed only in CD109+CECs+ but not in CD146+CECs.

### CD109+ and CD146+ CEC phenotype

**Figure 3:**
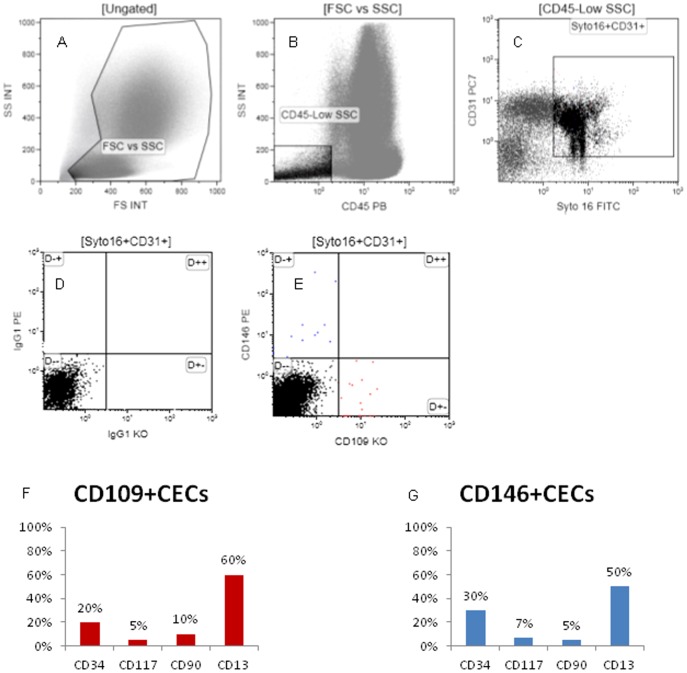
Flow cytometry strategy to detect CD109+CEC and CD146+CEC phenotype. After exclusion of debris (A) and selection of CD45− (B), nucleated (Syto16+) and CD31+ cells (C), CEC were identified as positive for CD109 or CD146 (E). (D): negative control. CD109+ CECs and CD146+CECs were evaluated by flow cytometry for the expression of CD34, CD117, CD90 and CD13.

As shown in Fig. 3, among Syto16+7-AAD-CD31+CD45− cells, we detected two different population of CECs, being one positive for CD109+ but negative for CD146, and one positive for CD146 but negative for CD109. Both CD146+ CECs and CD109+ CECs were negative for CD90 and CD117, to confirm these cells are likely differentiated cells and not progenitors. CD34 was positive on 30% of CD146+ CECs and on 20% of CD109+ CECs, and CD13 was positive on 50% and 60% of CD146+CECs and CD109+CECs respectively, confirming these cells are activated cells of angiogenic vasculature [44].

**Figure 4:**
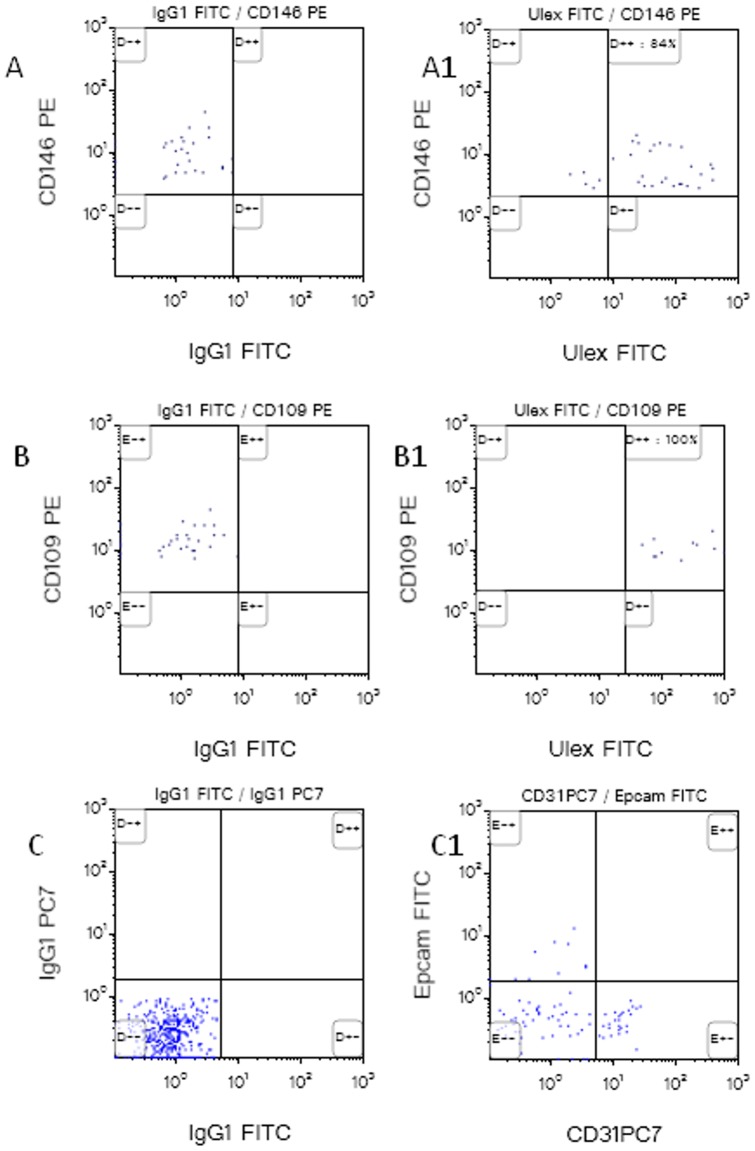
Expression of Ulex Europeaus Lectin.on CD109+CECs and CD146+CECs+ (4A–B1). Epcam staining (4C–C1) confirmed that Epithelial Cells even if present in the DNA+CD45− cell compartment, are negative for the expression of CD31 present only on endothelial cells.

Both CD146+CECs and CD109+CECs were positive for the Ulex Europeaus Lectin (Fig. 4 A–B1). To exclude epithelial cells contamination, we stained nucleated (Syto16+) CD45− cells for Epcam and even if a subpopulation of Epcam+ cells is present, these cells are negative for CD31 expression (Fig. 4 C–C1).

**Figure 5:**
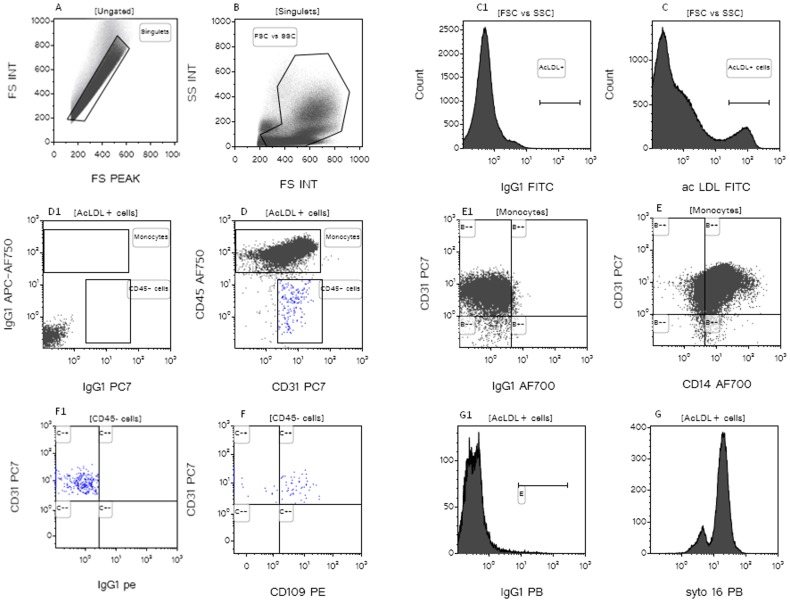
Flow cytometry strategy to detect Ac-LDL uptake. After dublets (A) and debris (B) exclusion, Ac-LDL+ cells were detected (C). Among these cells, CD45+CD31+ and CD45−CD31+ cells were detected (D). CD45+CD31+ were also positive for CD14 (monocytes,E) and CD45−CD31+ were also positive for CD109+ (endothelial cells, F). Ac-LDL+ cells were nucleated (Syto16+, F). C1, D1, E1 and F1 are the negative controls.

To examine CD109+CECs function, we performed Ac-LDL up-take. As Ac-LDL is taken up by macrophages/monocytes, we used these cells as “internal positive control” to confirm endocytosis activity. As reported in Fig. 5, among Ac-LdL+ cells both CD45+CD31+CD14+ (monocytes) and CD45−CD31+CD109+ (endothelial cells) were detected.

### Detection of CD109+ CECs in Healthy Subjects and Cancer Patients

Patients characteristics are reported in Table 1.

**Table 1:** Patients characteristics.

**Glioblastoma Pts**	
Number	134
Median Age	53.8 (27–72)
Gender (F∶M)	74∶60
Time for second blood collection	3 months after treatment beginning.
Treatment	Bevacizumab and Irinotecan or Bevacizumab alone
**Breast Cancer Pts**	
Number	66
Median Age	55.5 (37–81)
Gender (F∶M)	66∶0
Receptor Status	ER neg PgR neg HER2 neg
Time for second blood collection	2 months after treatment beginning.
Treatment	Metronomic Oral Chemotherapy with Cyclophoshamide+Capacetabine+Bevacizumab+Erlotinib

Significantly higher levels of CD109+CECs, CD146+CECs, viable CD109+CECs and viable CD146+CECs (P = 0.0001) were found at baseline in breast cancer patients and glioblastoma patients compared to healthy controls (Table 2). CD109+CECs were 26±20/mL in healthy subject (n = 50), 62±43/mL in metastatic breast cancer patients (n = 66, p<0.0001) and 101±84/mL in glioblastoma patients (n = 134, p<0.0001) before treatment. The fraction of apoptotic/necrotic CD109+CECs was 71±18% in healthy subject, 51±18% in breast cancer patients and 60±21 in glioblastoma patients.

**Table 2:** Baseline values of CD109+ CECs and CD146+ CECs.

	CEC109/mL	% Apoptotic CEC109	Viable CEC109/mL	CEC146/mL	% Apoptotic CEC146	Viable CEC146/mL
Healthy Subject	26±20	71±18	8±8	43±23	67±20	13±10
Breast Cancer Pts	62±43	51±18	30±23	103±83	62±20	43±49
Glioblastoma Pts	101±84	60±21	50±68	117±87	66±18	36±23

After treatment CD109+CECs and viable CD109+CECs were 52±42/mL and 15±20/mL respectively in breast cancer patients, and 64±60/mL and 35±30/mL in glioblastoma patients (Table 3).

**Table 3:** Viable CD109+ CECs and CD146+CECs before and after treatment.

	Viable CD109+ CECs		Viable CD146+ CECs	
	Before Treatment	After Treatment	Before Treatment	After Treatment
Breast Cancer Pts	30±23	15±20	43±49	41±88
Glioblastoma Pts	50±68	35±30	36±23	23±31

CD146+CECs were 43±23/mL in healthy subject (n = 50), 103±83/mL in breast cancer patients (n = 64, p<0.0001) and 117±87 in glioblastoma patients (n = 134, p<0.0001).

The fraction of apoptotic/necrotic CD146+CECs was 67±20% in healthy subject, 62±20% in breast cancer patients and 66±18/mL in glioblastoma patients before treatment.

After treatment CD146+CECs and viable CD146+CECs were 96±121/mL and 41±88/mL respectively in breast cancer patients, and 78±84/mL and 23±31/mL in glioblastoma patients (Table 3).

## Discussion

Endothelial cell heterogeneity has been described at the level of cell morphology, function, gene expression, and antigen composition. Because blood vessels are distributed throughout the body, the endothelial cells are exposed to an enormous variety of tissue microenvironments, and the wide range of signal inputs is sufficient to generate phenotypic heterogeneity across the vascular tree [19], [21], [35].

Detection of circulating endothelial cells by flow cytometry is an approach widely used in cancer patients, and the identification of the number, viability and kinetic of endothelial cells is a promising tool to stratify patient receiving anti-angiogenic treatment [15]–[18].

Currently, CECs are enumerated by a multiparametric flow-cytometry approach combining CD146, CD31, CD45, a nuclear binding antigen (Syto16, Dapi or HOECST) along with a viability cellular staining (like 7-AAD or Annexin V).

CD109 is a glycosylphosphatidylinositol-anchored cell surface glycoprotein and is one of 12 endothelial markers over-expressed in tumor endothelial cells [23], [24].

In this work, following an approach recently validated in our laboratory [14], we sorted CD109+CECs and CD146+CECs and we confirmed by RT-PCR, in both population the expression of mRNA level of CDH5 (Ve-Cadherin) and CLDN5 (Claudin5) two genes selectively expressed in endothelial cells. Higher levels of CLEC14a, TMEM204, ARHGEF15, Gpr116 expressed in developing endothelial cells [33], were present in CD109+CECs when compared to CD146+CECs, suggesting that these genes may play an important role not only in developing endothelial cells but also in adult angiogenesis.

We demonstrated that CD109+CECs and CD146+CECs are two different subpopulation of endothelial cells, being these two antigens not expressed on the same cells, while CD34 (a marker currently used to identify progenitor cells) was positive on 20% of CD109+CECs and on 30% of CD146+CECs.

We evaluated the number, viability and kinetic of CD109+CECs and of CD146+ CECs and observed that both subpopulation of endothelial cells are enriched in the blood of glioblastoma and breast cancer patients, are more viable when compared to value observed in healthy subject, and their number decrease significantly after treatment.

In our previous experience, we reported that in patients with breast cancer treated with metronomic chemotherapy, CD146+CEC levels after two months of treatment were associated with prolonged PFS [15]. In another clinical trial, in breast cancer patients treated with metronomic chemotherapy and bevacizumab, baseline CEC levels were also associated with PFS [36], [37] confirming that the quantification of CD146+ CECs is useful to identify patients who might benefit from antiangiogenic treatments [38].

In a series of patients with glioblastoma treated with AZD2171, a pan-VEGF receptor tyrosine kinase inhibitor, Batchelor found that viable CEC levels were higher in patients who experienced disease progression during AZD2171 therapy compared to patients without progression at day 112 [39].

We recently reported [40] that in glioblastoma patients treated with bevacizumab and irinotecan or with bevacizumab alone, PFS and OS were significantly increased in patients with baseline counts of CD109+CECs higher than 41.1/mls, (1stquartile), while no prognostic factor was associated to CD146+CECs.

These data suggest that CEC heterogeneity may reflect vascular tumor complexity and that CD109+ CECs might be a potentially useful prognostic marker for glioblastoma patients.

It has been reported that CD109 is over-expressed in tumor vasculature [24], and in this study we observed that all endothelial colonies from healthy subjects and from 10 cancer patients were positive for CD31, CD146, CD34 and VEGFR-2 and negative for CD45, while CD109 was positive on 1/9 endothelial cultures from healthy subject (11%) and on 5/10 (50%) endothelial cultures from cancer patients.

Moreover m-RNA level of TEM8, a cell surface glycoprotein identified as Anthrax Toxin Receptor 1, were detectable in CD109+CECs but not in CD146+CECs. It has been reported that TEM8, is up-regulated in tumor vessels [41], [42]. In TEM8 Knockout mice physiological angiogenesis and wound healing occur normally, but in tumor bearing mice, tumor growth is impaired, showing that TEM8 may be required to promote tumor angiogenesis but not normal development [43].

Taken together these results suggest that CD109 could represent a rare population of circulating tumor endothelial cells, that play a potentially useful prognostic role in patients with glioblastoma. The role of CD109 expression in cancer vessel-specific endothelial cells deserves to be further investigated by gene expression studies.

## Conclusions

There is a growing need in identifying circulating biological marker to stratify patients who may benefit of anti-angiogenic treatment. In this work we report that CD109 is a potential useful prognostic marker in glioblastoma patients, and that its role in cancer angiogenesis need to be investigated in different tumor setting.
